# Long‐Term Outcomes of Single‐Implant Mandibular Overdentures: A 8–10 Year Prospective Study Across Two Cohorts

**DOI:** 10.1111/clr.70065

**Published:** 2025-10-23

**Authors:** Xotchil Lourdes Tellez Flores, Lays Noleto Nascimento, Thalita Fernandes Fleury Curado, Túlio Eduardo Nogueira, Murali Srinivasan, Cláudio Rodrigues Leles

**Affiliations:** ^1^ School of Dentistry Federal University of Goias Goiania Brazil; ^2^ Clinic of General‐, Special Care‐ and Geriatric Dentistry, Center for Dental Medicine University of Zurich Zurich Switzerland; ^3^ Department of Reconstructive Dentistry, Division of Gerodontology School of Dental Medicine of the University of Bern Bern Switzerland

**Keywords:** cohort study, dental implant, edentulous patient, overdenture

## Abstract

**Objectives:**

This study aimed to evaluate the long‐term outcomes of two prospective cohorts of edentulous individuals treated with single implant mandibular overdentures (SIMO) opposed by a conventional maxillary complete denture.

**Material and Methods:**

A total of 74 participants, treated between 2013 and 2016 at the School of Dentistry of the Federal University of Goiás, Brazil, were included. A single midline implant was placed in the edentulous mandible; 93.2% of implants were immediately loaded, and outcomes were followed for 8–10 years. Data collected included dental patient‐reported outcomes (dPROs), implant survival, implant stability, peri‐implant health, and incidence of prosthodontic events. The frequency of the prosthetic maintenance and complication events was reported in terms of cumulative incidence. Within‐group longitudinal changes were tested using paired comparison tests. A significance level of *p* < 0.05 was used for statistical inferences.

**Results:**

Significant improvements in OHIP‐Edent and satisfaction scores were observed immediately post‐treatment and maintained throughout the follow‐up period, confirming a significant positive effect of SIMO on these outcomes (*p* < 0.001). The overall implant survival rate was 95.6%. Peri‐implant outcomes showed favorable health status, with mild bone loss observed (−1.55 ± 1.09 mm at the 8–10‐year follow‐up). Prosthetic complications and the need for recall visits for maintenance were common. The most frequent prosthetic complications were matrix replacement (43.3%) and overdenture fracture (12.4%).

**Conclusions:**

SIMO demonstrated sustained long‐term effectiveness with regular clinical monitoring and frequent maintenance. For most patients, the positive initial outcomes continued for up to 10 years, suggesting SIMO particularly benefits older patients with financial constraints.

## 1 Introduction

Today, implant therapy offers more cost‐effective solutions, such as implant overdentures (IODs), which enhance the stability and retention of conventional complete dentures for edentulous individuals. These advancements have demonstrated long‐term success and have significantly improved quality of life. Older edentulous adults particularly benefit from IOD therapy, as it generally involves conservative surgical techniques, requires fewer implants, and aligns well with the concept of reducing treatment burden (Schimmel et al. 2017).

A single midline implant was first proposed in 1997 (Cordioli et al. 1997), aiming to provide a minimally invasive and a more affordable treatment option for the edentulous older adult. Single implant mandibular overdentures (SIMO) have been shown to enhance masticatory efficiency (Nogueira et al. 2017; Coutinho et al. 2022; Passia and Kern 2023; Liddelow et al. 2024), achieving high patient satisfaction and favorable short‐term implant survival rates when compared to two‐implant IODs (Bryant et al. 2015; Hartmann et al. 2020a, 2020b; de Resende et al. 2021; Srinivasan et al. 2016). Long‐term studies on SIMO have further confirmed a high implant survival rate, with increased patient satisfaction and a significant increase in all comfort and functional parameters with few reported complications (Passia et al. 2019; Liddelow et al. 2024; Yazigi et al. 2025).

However, there is a significant lack of long‐term follow‐up studies in the scientific literature that involve larger sample sizes, multiple implant and retention systems, and examine a comprehensive set of core outcomes, including peri‐implant tissue health, complication‐free survival rates for both implants and prosthetics, and overall impact on quality of life (Tonetti et al. 2023).

The present study reports on long‐term outcomes, up to 10 years of follow‐up, with SIMO examining a comprehensive set of core outcomes. This study aimed to enhance the existing evidence on SIMO by evaluating the clinical, radiographic, and dental patient‐reported outcomes in two prospective cohorts of edentulous individuals rehabilitated with a single‐implant mandibular overdenture, opposed by a conventional maxillary complete denture, over a follow‐up period of 8 to 10 years.

## 2 Materials and Methods

This prospective cohort study has been reported in accordance with the STROBE (Strengthening the Reporting of Observational studies in Epidemiology) guidelines (Von Elm et al. 2007). The study protocol of the long‐term follow‐up was approved by the local Research Ethics Committee (CAAE: 74402323.8.0000.5083), and an informed signed consent was collected from all participants. Participants (*n* = 74) in this study were from two cohorts treated with SIMO between 2013 and 2016 at the School of Dentistry of the Federal University of Goiás, Brazil (Nogueira et al. 2018, 2021). Participants were included if they were: edentulous and were using conventional maxillary and mandibular dentures, with satisfactory general health, and adequate bone available in the symphyseal region of the mandible for the placement of an implant of at least 9 mm in length (Nogueira et al. 2018; Coutinho et al. 2022). Exclusion criteria for the participants included: severe cognitive decline, and subjects who were not willing for SIMO therapy.

All participants received 3.75 mm diameter external hexagon implants (Titamax TI Cortical, Neodent, Brazil) placed in the mandibular midline using a single‐stage, minimally invasive surgery protocol. Immediate loading was applied when the insertion torque exceeded 30 Ncm and the implant stability quotient (ISQ) was above 60. Immediate loading was done in 69 participants (93.2%), while 5 participants (6.8%) underwent delayed loading. For overdenture retention, a titanium ball‐type abutment was used, along with a nylon matrix and capsule (O‐ring attachment, Neodent, Brazil). No type of denture reinforcement, such as a cast framework or fiber reinforcement, was used in the construction of the SIMO. Detailed surgical and prosthetic procedures are detailed in former publications (Nogueira et al. 2018).

The various outcomes assessed at different time points are detailed in Table 1. Participants were clinically assessed at the scheduled time points for the various outcomes throughout the entire follow‐up period. Final data collection took place between September 2023 and July 2024, including the following outcomes: dental patient‐reported outcomes, implant survival, implant stability, peri‐implant health status, and incidence of prosthodontic events. The treatment endpoint at each of the follow‐up periods was classified according to the six‐field protocol criteria described by Payne et al. (2001):

1. Successful—no evidence of retreatment except for acceptable maintenance interventions: patrix/matrix activation or replacement, and/or asymptomatic, peri‐implant mucosal enlargement, not requiring excision; no more than two patrix/matrix replacements/adjustments in the first year, and over 5 years a maximum of five replacements and no more than one reline of the overdenture base
2. Surviving—the participant attended the recall visit or, even if not examined directly, confirmed no evidence of retreatment, except that described for a successful outcome
3. Unknown (lost to follow‐up)—the patient cannot be traced; surviving or successful implant overdenture removed to allow the provision of a new overdenture with another overdenture design with additional implants or a fixed implant prosthesis using the same or additional implants
4. Dead—the patient died during the study period, regardless of whether successful or surviving criteria were experienced before death
5. Retreatment (repair)—Treatment of the implant overdenture and/or mucosa, where the marginal integrity and associated patrices/matrices are maintained irrespective of modifications as long as it continues as an implant overdenture; more than two replacements of either patrix or matrix in the first year or more than five in the first 5 years; and includes replacement of worn or fractured overdenture teeth, fractured overdentures, relining of the overdenture more than once in 5 years, or excision of patrix associated with mucosal enlargement as a result of infringement on the shoulder/undersurface of the patrix;
6. Retreatment (replacement)—part or the entire overdenture is no longer serviceable due to implant loss or irreparable mechanical breakdown; a replacement prosthesis was indicated.

**TABLE 1:** Outcome data collected for each cohort, according to the study time points.

Outcomes	Time‐points							
	Baseline	3‐month	6‐month	1‐year	2‐year	5‐year	8‐year	10‐year
OHIP‐Edent	□■	□	□■	□■	□	□	■	□
Satisfaction	□■	□	□■	□■	□	□	■	□
Implant stability quotient (ISQ)	□■	□	□■	□■	□	□	■	□
Sulcus bleeding	□■	□	□■	□■	□	□	■	□
Suppuration	□■	□	□■	□■	□	□	■	□
Plaque	□■	□	□■	□■	□	□	■	□
Calculus	□■		□■	□■			■	
Peri‐implant mucosal level	□■	□	□■	□■	□	□	■	□
Peri‐implant bone level	□■		□■	□■			■	
Prosthetic complications	□■	□■	□■	□■	□■	□■	□■	□■
Implant survival	□■	□■	□■	□■	□■	□■	□■	□■

*Note:* □ Cohort #1 – Implant placement in the year 2013. ■ Cohort #2 – Implant placement in the year 2015–2016.

Dental patient‐reported outcomes (dPROs) included patient denture satisfaction assessed with a Visual analog scale (VAS) ranging from 1 (not satisfied) to 100 (completely satisfied) for overall satisfaction, comfort, stability, ability to chew and speak, and aesthetics (Nogueira et al. 2017). Oral Health‐Related Quality of Life (OHRQoL) was assessed using the Brazilian version of the Oral Health Impact Profile for the Edentulous (OHIP‐Edent) (Souza et al. 2010).

The implant stability quotient (ISQ) was measured using a resonance frequency analysis device (Osstell AB, Göteborg, Sweden). Soft tissue conditions were assessed using the sulcus bleeding index (Mombelli et al. 1987) and plaque index (Silness and Löe 1964). The distance between abutment platforms and the coronal peri‐implant mucosal margin was measured using a periodontal probe (Colorvue, Hu‐Friedy UNC 12).

Peri‐implant marginal bone levels were evaluated using digital periapical radiographs with a Scan eXam digital phosphate plate system (KaVo Dental GmbH, Biberach, Germany), with a modified anterior film holder to ensure precision obtained at baseline, 1 year, and 8–10 years after implant loading. Using a standardized protocol, measurements were then analyzed with Cliniview 10.2.6 (DEXIS, Washington, D.C., USA).

Throughout the entire follow‐up period, prosthodontic complications were documented including: component replacements, repairs, substitutions of the denture base (such as addressing fractures, relining, or rebasing), and the fabrication of new dentures (when needed). Incidence rates were calculated accordingly.

Data were summarized, and the Shapiro–Wilk test was used to confirm the normality of the data. Bivariate pairwise comparison tests were used to assess the longitudinal changes in outcome parameters. All data analyses were conducted using IBM SPSS Statistics (IBM SPSS Statistics, v24.0, Armonk, NY, USA) at a significance level of *p* < 0.05.

## 3 Results

**FIGURE 1:**
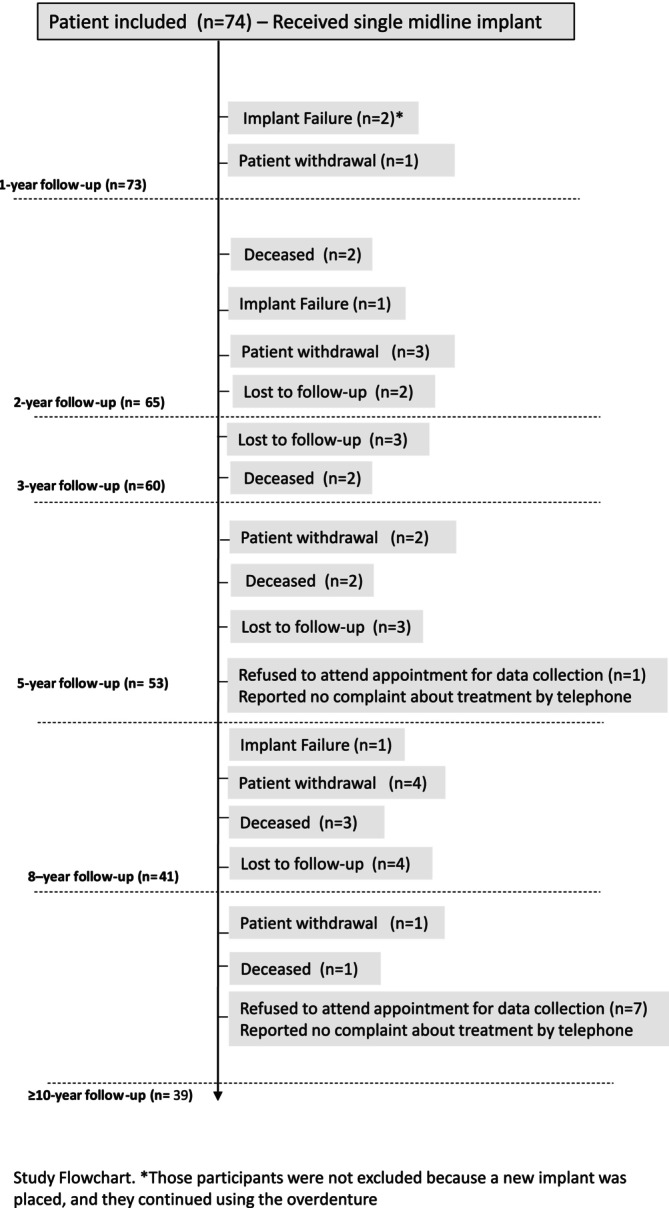
Patient flowchart.

A total of 74 patients entered this study and were submitted to implant surgery for placement of a single midline implant in the edentulous mandible. Fifty‐four (73%) were female, and their ages ranged from 47 to 86 years (mean = 64.7 ± 8.9). Overall, the combined initial sample size included participants from cohort #1 (*n* = 42) and cohort #2 (*n* = 32). The complete patient flowchart is detailed in Figure 1.

**FIGURE 2:**
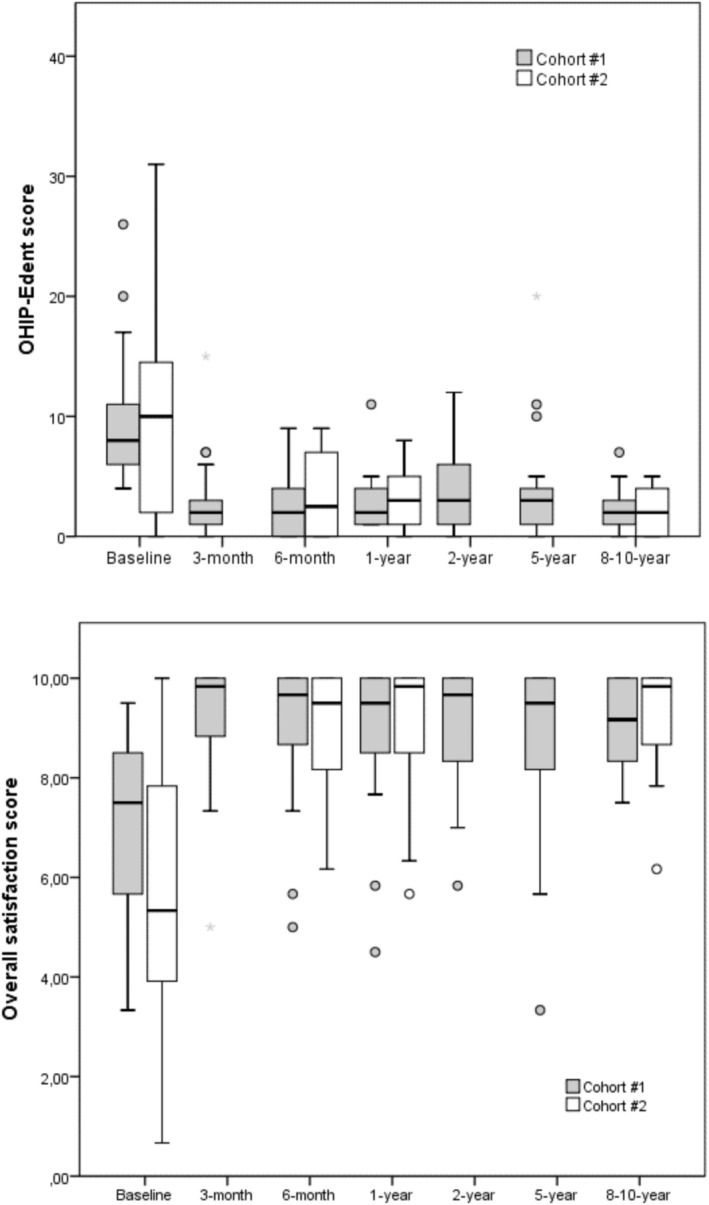
Distribution of data on dental patient‐reported outcomes—OHIP‐Edent (upper) and overall satisfaction score (lower), according to the study time points and patient cohort.

The changes in dental patient‐reported outcomes are shown in Figure 2. No significant effect of the two different cohorts was observed for all tested outcomes. On the other hand, significant improvement from baseline was observed for the OHIP‐Edent (reduction in scores) in the immediate follow‐up, sustained throughout the entire period until the last follow‐up (*p* < 0.001; Effect size = 0.95). Similarly, the satisfaction scores increased significantly from baseline and sustained until the end of the study (*p* < 0.001; Effect size = 0.77).

The descriptive data on peri‐implant outcomes are detailed in Table 2. A significant reduction in bleeding index (*p* < 0.001) and significant marginal bone loss (*p* < 0.001) were observed. The mean reduction in peri‐implant bone levels was −0.68 ± 0.94 mm after the first year and −1.55 ± 1.09 mm at the last follow‐up (8–10 years) (*p* < 0.001).

**TABLE 2:** Summary data on the peri‐implant outcomes.

Time point	Bleeding	Plaque	Distance (mm)*	Marginal bone level (mm)		Implant stability (ISQ)
				Mean (SD)		
				Bone level	Change from baseline	
	*n* (%)	Median (IQR)	Mean (SD)			Mean (SD)
Baseline	24 (32.4)	1.00 (2)	−0.034 (0.85)	−0.94 (0.76)	Reference	75.0 (7.9)
3 months	14 (34.1)	2.00 (1)	−0.057 (0.09)	—	—	73.4 (4.9)
6 months	12 (19.0)	2.00 (1)	−0.106 (0.08)	—		77.4 (4.7)
1 year	9 (17.0)	1.00 (2)	−0.127 (0.07)	−1.62 (0.78)	−0.68 (0.94)	79.2 (4.1)
2 years	3 (8.1)	2.00 (2)	−0.119 (0.07)	—	—	78.7 (4.4)
5 years	2 (5.1)	1.00 (1)	−0.172 (0.08)	—	—	80.4 (3.8)
8–10 years	4 (13.8)	1.00 (2)	−0.211 (0.10)	−2.49 (1.02)	−1.55 (1.09)	72.6 (5.8)
*p* **	< 0.001	0.918	< 0.001		< 0.001	0.047

* From the implant platform to the peri‐implant mucosal margin (mm).

** Paired comparison between baseline and last follow‐up.

The cumulative incidences of prosthetic maintenance or complication events are detailed in Table 3. The most frequent complication was the need for matrix replacement (43.3%), followed by overdenture fracture (12.4%). Considering the possible occurrence of multiple events per patient and the different follow‐up times, the incidence density rates (or person‐time incidence rates) were calculated by taking the total number of new cases of the event and dividing that by the sum of the person‐time of the at‐risk population. In this study, the follow‐up time of all included subjects, including censored cases, ranged from 1 to 11 years, summing a total of 473.4 years. Therefore, the higher incidence density rates were for matrix replacement (55.7% of patients at risk with an event per year), followed by overdenture fracture (15.6% of patients at risk with an event per year).

**TABLE 3:** Frequency of the prosthetic maintenance and complication events (*n* = 598).

	Frequency	Percent	Incidence density	Incidence density (%)
Matrix replacement	259	43.3	0.55	54.7
Overdenture fracture	74	12.4	0.16	15.6
Attachment replacement	45	7.5	0.10	9.5
Matrix + housing replacement	42	7.0	0.09	8.9
Attachment loosening	39	6.5	0.08	8.2
Laboratory relining	33	5.5	0.07	7.0
Teeth fracture/repair	31	5.2	0.07	6.5
Direct relining	28	4.7	0.06	5.9
New overdenture	22	3.7	0.05	4.6
Replacement of the retention system	16	2.7	0.03	3.4
Soft tissue surgery	9	1.5	0.02	1.9

Finally, the final treatment endpoint was assessed and reported according to the number of patients at risk in each of the time points of the study, and is detailed in Table 4. The overall failure rate was 17.6% (*n* = 13), which included replacing treatment with additional implants (*n* = 12), allowing one patient to withdraw and return to the conventional denture. The cases classified as “retreatment–repair” were only identified in the first year of follow‐up, according to the classification criteria. Moreover, at the 8–10‐year follow‐up, 39 patients were identified as continuing with the SIMO treatment, including both successful and surviving cases.

**TABLE 4:** Overall final SIMO treatment outcome according to the time point and the number of patients at risk.

Time period	Up to 1‐year	> 1 and < 8 years	8–10 years
Number of patients at risk*	74	73*	41*
Outcome			
Successful	66 (89.2)	40 (54.8)	32 (78.0)
Retreatment (repair)	7 (9.5)	n/a	n/a
Surviving	0	1 (1.4)	7 (17.1)
Unknown (lost to follow‐up)	0	12 (16.4)	0
Dead	0	9 (12.3)	1 (2.4)
Retreatment (replacement)	1 (1.4)	11 (15.1)	1 (2.4)

Abbreviation: n/a, not applied.

* Excluding unknown, dead, and retreatment (treatment withdrawal).

## 4 Discussion

This prospective study reports the 8 to 11‐year long‐term outcomes of patients treated with a SIMO with opposing conventional maxillary complete dentures. Overall, SIMO provided reliable outcomes regarding dental patient‐reported outcomes, favorable soft tissue conditions, and secondary implant stability throughout the follow‐up period. Furthermore, although the implant survival rate was high, the occurrence of prosthodontic complications and the need for regular maintenance were common, especially the need for matrix replacement. On the other hand, some patients did not adapt to SIMO treatment, and the occurrence of deviation from the original treatment protocol occurred in 12 patients. Therefore, the findings validated the hypothesis that a SIMO can achieve enduring and positive outcomes; nevertheless, the variability in maintenance events among patients highlighted the diverse ongoing care needs, and regular recall visits should be scheduled due to frequent maintenance interventions (Passia et al. 2019; Yazigi et al. 2025).

There are few long‐term studies on the longitudinal performance of SIMO with limited sample sizes (Liddelow et al. 2024; Yazigi et al. 2025). Yazigi et al. (2025) reported a prospective pilot study with 11 patients followed for up to 15 years (*n* = 5 at the last follow‐up). They found a high implant survival rate (100%), and the most common complications were the incidence of overdenture fractures (*n* = 8), and the most frequent prosthetic maintenance event was the activation of the matrix due to loss of retention and exchange of the female part. Another study (Liddelow et al. 2024) followed 29 SIMO patients for up to 15 years (*n* = 14 at the last follow‐up), with 100% implant survival and high patient satisfaction, with a significant increase in all comfort and functional parameters. In summary, these two long‐term studies concluded that SIMO is a safe, reliable, and cost‐effective treatment with high levels of patient satisfaction long term despite the need for regular maintenance visits (Yazigi et al. 2025; Liddelow et al. 2024).

The overall implant survival rate in this study was 95.6%. Among the 73 patients, three of them in which the immediate loading protocol was used experienced implant complications. One lost their implant after 21 months and switched to a conventional mandibular complete denture. Two other patients required implant replacements due to peri‐implant complications within the second year but continued with follow‐up. One of these patients unfortunately passed away during the sixth year, while the other completed the full 8‐year follow‐up (the initial implant was placed in 2013, and the replacement took place in 2015). These findings underscore the efficacy of the immediate loading protocol, but also highlight the potential for complications and the need for ongoing patient management. Immediate loading protocols have been associated with survival rates ranging from 84.7% to 100%, whereas delayed loading protocols show survival rates between 90.9% and 100% (Kern et al. 2021; Passia and Kern 2023). Kern et al. (2021) investigated whether the loading protocol of single dental implants placed for single‐implant mandibular overdentures (SIMO) influences implant survival. Based on data from 102 participants enrolled in a 5‐year randomized, multicenter clinical trial, a statistically significant lower implant survival rate was observed in the immediately loaded group compared to the conventionally loaded group (84.7% vs. 96.2%). Therefore, the authors recommended that immediate loading should be considered only in exceptional cases (Kern et al. 2021). Although the survival rate in the current study is deemed acceptable, it is advisable to use immediate loading protocols with caution and tailor them to individual patient requirements.

Compared to the baseline, there was a significant improvement in OHRQoL, as evidenced by the decrease in OHIP‐Edent scores (Nogueira et al. 2024). These lower scores were consistently maintained throughout the longitudinal assessment, with no significant differences observed between time points. Satisfaction with the maxillary denture remained relatively stable compared to baseline, with no significant differences found between periods. In contrast, satisfaction with the mandibular overdenture showed a substantial improvement from baseline, and this enhanced level of satisfaction was sustained throughout the follow‐up period, with no significant differences detected between time points.

The study also highlights the variability in maintenance requirements among individuals undergoing mandibular overdenture treatment. The most common maintenance event was the matrix replacement (*n* = 259), followed by adjustment of the retentive elements (*n* = 219). Previous research suggests that maintenance events in SIMO, such as matrix replacements, typically occur annually to maintain proper retention (de Araújo et al. 2022). Nonetheless, the frequency of such events may vary depending on individual patient factors as well as on the type and quality of the attachment system used. Certain attachments, when worn out, require replacement, as observed in the present study. In contrast, others may have their retention restored simply through reactivation, as reported by Passia et al. (2019). Therefore, depending on whether replacement or reactivation is required, as well as the longevity of the attachment system, the cost associated with maintenance procedures may vary.

Accepted guidelines generally recommend no more than two matrix replacements and no more than one reline in the first year, with a maximum of five over 5 years to optimize performance (Payne et al. 2001). Regarding the number of relines, although this is a widely accepted guideline, considering a treatment as “successful” only if a single reline is performed within a 5‐year period may not be reasonable in the context of overdentures. It is well established that overdentures may require more frequent relines than conventional complete dentures, for which the five‐year reline interval is a stronger recommendation. In overdenture cases, additional relines may be necessary due to factors such as the need for reintegration of the retention system or adjustments following mechanical complications. These interventions, when properly managed, do not necessarily indicate treatment failure and may, in fact, reflect appropriate maintenance within a successful therapeutic outcome.

The study also identified midline fractures as a common complication associated with oral function denture dropping, which are frequently associated with contributing factors such as mechanical stress, altered force distribution, and techniques used for integrating retentive inserts into the denture and material issues (de Paula et al. 2020). Therefore, regular maintenance visits are essential for managing complications effectively and enhancing the long‐term functionality of prostheses. The need for relining was evaluated during the follow‐up period, and overdentures that showed signs of instability were relined. Furthermore, any significant functional issues reported by the patient and confirmed clinically were addressed promptly to maintain proper denture performance.

One important limitation of this study is that the reduction in sample size due to participant deaths and loss of follow‐up, common in long‐term studies, could affect result reliability. The occurrence of missing data is a common limitation in longitudinal studies due to participant attrition, non‐response, or other factors leading to incomplete data collection across multiple time points. Missing data can reduce statistical power, introduce bias in results and interpretation, and complicate data analysis, especially if missing data is not random, which is a common factor related to patient dissatisfaction with treatment and withdrawal from the study. Therefore, there is a risk of overestimation of the true treatment effects due to missing data because of attrition bias.

Though substantial, the study's follow‐up duration may not capture complications emerging beyond this period. Extended follow‐up in future research could offer deeper insights into the durability and sustainability of SIMO. Additionally, while the study showed improvements in OHRQoL and satisfaction, variations in individual maintenance needs and prosthesis functionality could influence outcomes and are not fully addressed by the study. Moreover, although a cost analysis was not within the scope of this study, it is reasonable to assume that, over time, the cumulative cost of frequent maintenance procedures—such as replacement or reactivation of attachment components—may approximate or even exceed the cost of placing a second implant, particularly in cases requiring multiple interventions. Previous data from a randomized clinical trial (Hartmann et al. 2020a, 2020b) suggested that, within 1 year of mandibular overdenture use, whether supported by one or two implants, this approach is more cost‐effective than fixed implant treatment for the edentulous mandible. Hence, although cost is not the only factor to be considered when determining the most appropriate treatment approach, it remains an important aspect of the decision‐making process for dentists and other stakeholders involved in the development and implementation of health policies.

The study reports on several variables: patient satisfaction, OHRQoL, and maintenance requirements. The broad scope of analyses increases the complexity of interpreting results and requires careful consideration of whether findings across different measures align consistently. For example, regarding maintenance events, the substantial number of matrix replacements and fractures observed in the present study may appear higher than those commonly reported in studies involving two‐implant mandibular overdentures (TIMO). A randomized clinical trial comparing mandibular overdentures retained by one or two implants showed that the two treatments performed similarly when considering the incidence of fractures and the need for prosthodontic maintenance, including adjustments of the overdenture and the attachment system (de Resende et al. 2023). However, it is important to consider the ongoing debate concerning the impact of implant number on maintenance outcomes of mandibular overdentures. A recent umbrella systematic review (Milić Lemić et al. 2025), which included seven systematic reviews, presented evidence favoring SIMO. Some included studies reported higher prosthetic failure rates for TIMO, while others found no significant differences in maintenance outcomes when compared to SIMO. Two systematic reviews (Alqutaibi et al. 2017; de Souza Batista et al. 2018) analyzing long‐term follow‐up data reported no significant differences between SIMO and TIMO in terms of prosthetic maintenance needs, including relining, occlusal adjustments, or replacement of attachment components. These findings suggest that the maintenance demands of SIMO are not necessarily greater—and may, in some cases, be lower—than those of TIMO, reinforcing the clinical viability of SIMO, especially for elderly or cost‐sensitive populations (Milić Lemić et al. 2025).

While the consensus previously favored using two implants for mandibular overdentures (Feine et al. 2002; Thomason et al. 2009), studies suggest that single‐implant overdentures can be a viable and cost‐effective option for edentulous patients (Hartmann et al. 2020a, 2020b; Passia and Kern 2023). Research indicates that single‐implant overdentures can achieve satisfactory outcomes in specific cases, offering an affordable alternative without compromising effectiveness (Cordioli et al. 1997; Srinivasan et al. 2016; Nogueira et al. 2017; Passia et al. 2019; Hartmann et al. 2020a, 2020b; Coutinho et al. 2022). This evidence underscores the potential benefits of single‐implant overdentures in appropriate clinical contexts, and the study's findings were consistent with evidence suggesting that single‐implant overdentures opposed by a conventional complete denture can be a viable alternative.

## 5 Conclusions

Within the limits of this prospective study, it was concluded that SIMO presents long‐term effectiveness under the conditions of continued regular clinical monitoring and frequent maintenance. The positive outcomes achieved in the initial period of use after implant placement and loading were sustained up to 8–10 years of follow‐up for most patients. Since the implant intervention has minimal costs and low complexity, this alternative can be useful for older patients with financial constraints as a preferred option in some clinical settings.

## Author Contributions

**Xotchil Lourdes Tellez Flores:** investigation, writing – original draft, methodology, visualization, data curation, writing – review and editing. **Lays Noleto Nascimento:** investigation, writing – original draft, methodology, visualization, writing – review and editing, data curation. **Thalita Fernandes Fleury Curado:** investigation, writing – original draft, methodology, visualization, writing – review and editing, data curation. **Túlio Eduardo Nogueira:** conceptualization, investigation, writing – original draft, methodology, validation, writing – review and editing, data curation, supervision. **Murali Srinivasan:** writing – original draft, writing – review and editing, validation, supervision. **Cláudio Rodrigues Leles:** conceptualization, investigation, funding acquisition, writing – original draft, methodology, validation, writing – review and editing, formal analysis, data curation, supervision, resources.

## Ethics Statement

The clinical standards of this study followed the Declaration of Helsinki, a statement of ethical principles for medical research involving human subjects.

## Consent

The study protocol was approved by the research ethical committee of the Federal University of Goias (CAAE 74402323.8.0000.5083), and all participants were informed about it and signed an informed consent form to participate in the study.

## Conflicts of Interest

The authors declare no conflicts of interest.

## Data Availability

The data that support the findings of this study are available from the corresponding author upon reasonable request.
